# Electronic Nicotine Delivery Systems and Clinical Outcomes in Adults With Inflammatory Bowel Disease: A Systematic Review of Emerging Evidence

**DOI:** 10.7759/cureus.108605

**Published:** 2026-05-10

**Authors:** Hatem Ahmed, Motaz Almahmood, Alexandra Short, Sameh Gomaa, Khloud S Abdelrazeq, Aamir Shahzad, Ali Alharethi, Khaled Elsokary, Mohamed K Mansour, John Marshall

**Affiliations:** 1 Internal Medicine, Phoenixville Hospital-Tower Health, Phoenixville, USA; 2 Library Services, Reading Hospital-Tower Health, West Reading, USA; 3 Faculty of Medicine, Cairo University, Cairo, EGY; 4 Gastroenterology, Lehigh Valley Health Network, Allentown, USA; 5 Gastroenterology, McMaster University, Hamilton, CAN; 6 Gastroenterology, Reading Hospital-Tower Health, West Reading, USA; 7 Internal Medicine, Rochester Regional Health/Unity Hospital, Rochester, USA

**Keywords:** crohn’s disease (cd), electronic cigarettes' e-cigarettes' vaping' e-smoking, electronic nicotine delivery systems (endss), endoscopic disease activity, inflammatory bowel disease, postoperative recurrence, smoking cessation, smoking vs vaping, systematic review, ulcerative colitis (uc)

## Abstract

Electronic nicotine delivery systems (ENDSs), including e-cigarettes and vaping, are increasingly used by adults with inflammatory bowel disease, often during smoking cessation or transition from combustible cigarettes, yet their clinical implications remain uncertain. We conducted a systematic review following the Preferred Reporting Items for Systematic Reviews and Meta-Analyses 2020 statement. MEDLINE, Embase, Web of Science, and Scopus were searched from inception through December 18, 2025, together with grey literature sources; the search was rerun on April 24, 2026, identifying no additional eligible studies. Two reviewers independently screened studies and extracted data, and risk of bias was assessed using design-appropriate tools. Four observational studies involving 1,869 participants, of whom 178 were ENDS users, met inclusion criteria. In nonoperative settings, a matched case-control study found no association between current e-cigarette use and a composite outcome of biologic initiation or change, hospitalization, or surgery; smaller cohorts were imprecise. In postoperative Crohn’s disease, endoscopic recurrence at one year was higher among exclusive e-cigarette users than among nonsmokers, with a directionally elevated but statistically nonsignificant adjusted estimate and higher biochemical activity also observed. Available evidence does not demonstrate a consistent association between current ENDS use and major inflammatory bowel disease outcomes in nonoperative settings, but a possible signal exists for higher postoperative endoscopic recurrence among e-cigarette users. Larger prospective studies with standardized exposure definitions are needed.

## Introduction and background

Inflammatory bowel diseases (IBD), including Crohn's disease (CD) and ulcerative colitis (UC), are chronic inflammatory conditions characterized by a relapsing-remitting course that contributes substantially to global morbidity, healthcare utilization, and long-term disability [1]. Recent epidemiologic data demonstrate that IBD are now global diseases, with rising incidence and steadily increasing prevalence [2].

Cigarette smoking remains one of the most modifiable environmental exposures in IBD, with a divergent direction of association by phenotype. In CD, active smoking is consistently associated with worsening of the disease course and is a recognized risk factor for postoperative recurrence [3]. Major society guidance emphasizes counselling for smoking cessation as part of routine CD management [4,5]. However, in UC, there appears to be an inverse association between smoking and disease onset and severity of disease [6].

Against this backdrop, nicotine consumption patterns have shifted with widespread uptake of electronic cigarettes and related electronic nicotine delivery systems (ENDSs). In the United States, nationally representative survey data indicate that adult e-cigarette use increased from 4.5% in 2019 to 6.5% in 2023 [7].

ENDS use has been reported among individuals with IBD, often in the context of smoking cessation attempts or transition away from combustible cigarettes, and may therefore occur alongside prior or concurrent combustible cigarette exposure [8-10]. Unlike combustible cigarettes, ENDS aerosols contain variable combinations of nicotine, solvents (e.g., propylene glycol/vegetable glycerin), and flavoring chemicals that may influence the intestinal epithelial lining, including mucosal barrier integrity and downstream inflammatory signaling [11]. Experimental models suggest that chronic e-cigarette aerosol exposure can impair intestinal barrier markers and promote pro-inflammatory responses, including with nicotine-free formulations [11]. However, the clinical implications of ENDS use in IBD, particularly for disease activity, complications, and treatment escalation, remain unanswered due to limited clinical evidence.

Despite the clinical relevance of this question, no prior systematic review has specifically synthesized clinical outcome data related to ENDS use in adults with IBD, and postoperative CD recurrence, a context in which any tobacco-related exposure is particularly consequential, has not been examined in a dedicated synthesis. We therefore conducted a systematic review to synthesize the available clinical evidence on the association between ENDS (e-cigarette/vaping) exposure and IBD outcomes in adults, addressing two clinically relevant comparisons: (1) ENDS use versus no-ENDS controls (primary comparator), and (2) ENDS use versus combustible cigarette smoking (secondary comparator).

## Review

Methods

Protocol and Reporting

This systematic review was conducted in accordance with the Preferred Reporting Items for Systematic Reviews and Meta-Analyses (PRISMA 2020). A completed PRISMA 2020 checklist is provided in the Appendices. The protocol was developed a priori and registered in PROSPERO (CRD420251184953).

Literature Search

A comprehensive search was conducted in MEDLINE (PubMed), EMBASE (Elsevier, embase.com), Web of Science Core Collection (Clarivate), and Scopus (Elsevier). The search strategy was developed by a medical librarian in consultation with the clinical lead authors, using controlled vocabulary and keywords for IBD, ENDS, and targeted IBD outcomes. The primary strategy was developed for MEDLINE and translated for the other databases. No filters or limits were applied during the search stage. All databases were searched from their inception through December 18, 2025. ClinicalTrials.gov, Google Scholar and Mednar were searched for grey literature on December 18, 2025, using a combination of keywords. Book publication records were manually filtered from the Google Scholar search result records prior to importing the Google Scholar records into Covidence (Covidence Systematic Review Software, Veritas Health Innovation, Melbourne, Australia).

No language restrictions were applied at the search stage; non-English full texts were eligible and were assessed for inclusion when accessible. To ensure currency of the evidence, the literature search was rerun on April 24, 2026, applying the same strategies across all databases and grey literature sources; no additional eligible studies were identified beyond those captured in the original search. Full search strategies are provided in the Appendices.

Eligibility Criteria

Inclusion criteria comprised studies of adults (≥18 years) with IBD (CD and/or UC) reporting exposure to ENDSs, including e-cigarettes and vaping. Eligible study designs were observational clinical studies, including prospective or retrospective cohort studies, case-control studies, and cross-sectional analyses, that reported outcomes according to ENDS exposure status. Outcomes of interest included IBD disease activity, IBD-related hospitalization, IBD-related surgery, systemic corticosteroid therapy, and, where reported, biochemical markers such as fecal calprotectin or C-reactive protein. Exclusion criteria were non-human studies, studies evaluating combustible cigarette smoking only without an ENDS exposure group, reviews, editorials, commentaries, and case reports or case series without a comparator. Conference abstracts were excluded a priori because limited outcome ascertainment and covariate adjustment precluded reliable risk-of-bias appraisal and synthesis.

Study Selection

**Figure 1:**
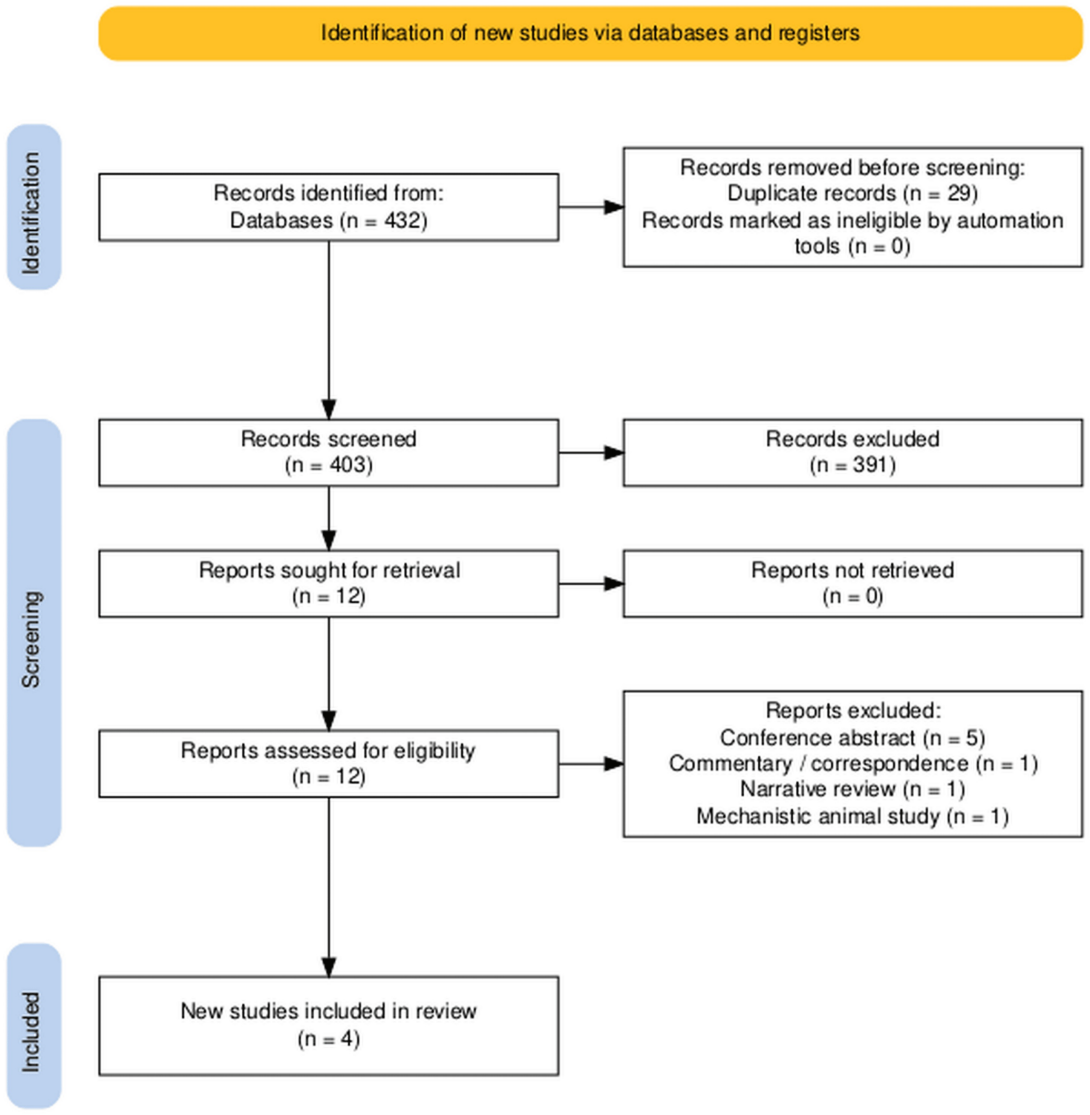
PRISMA flow diagram of study selection Flow of records through identification, screening, eligibility, and inclusion in the systematic review, prepared in accordance with the PRISMA 2020 statement. PRISMA: Preferred Reporting Items for Systematic Reviews and Meta-Analyses

All records were imported into Covidence for deduplication and screening. Two reviewers (HA, MA) independently screened titles and abstracts, followed by full-text review of potentially eligible articles. Discrepancies were resolved by discussion. When consensus could not be reached, a third reviewer (SG) adjudicated. Inter-rater reliability (Cohen's κ) was 0.67 for title and abstract screening (97% agreement) and 0.66 for full-text eligibility assessment (83.3% agreement), calculated prior to consensus or adjudication. Reasons for full-text exclusion were recorded and are presented in the PRISMA flow diagram (Figure 1).

Data Extraction

Using a standardized extraction form, two reviewers (HA, MA) independently extracted data. Extracted items included study identifiers (author, year, country), design and setting, sample size, IBD subtype, exposure and comparator definitions (including dual use and prior smoking history where reported), follow-up duration, outcome definitions and ascertainment, adjusted and unadjusted effect estimates, covariates included in multivariable models, and key limitations relevant to confounding and exposure misclassification. When outcomes were presented as percentages with available denominators, event counts were reconstructed by rounding (% × n) for exploratory analyses.

Risk-of-Bias Assessment

Two reviewers (HA, MA) independently assessed risk of bias for each included study using design-appropriate tools. Cross-sectional studies were evaluated using the Joanna Briggs Institute (JBI) analytical cross-sectional checklist. Cohort and case-control studies were evaluated using the Newcastle-Ottawa Scale (cohort and case-control versions, respectively). Discrepancies were resolved through discussion, with arbitration by a third reviewer (SG) when needed. Overall risk-of-bias judgments were considered during interpretation of findings, with particular attention to confounding by combustible cigarette smoking history and dual use, exposure ascertainment, outcome measurement, and imprecision in strata with small numbers of ENDS users.

Statistical Analysis and Data Synthesis

We assessed the feasibility of meta-analysis a priori; however, quantitative pooling was not performed because the included studies were clinically and methodologically heterogeneous. Findings were instead synthesized narratively by disease subtype and clinical context.

For dichotomous outcomes, we preferentially extracted adjusted odds ratios (aORs) and 95% confidence intervals (CIs). When adjusted estimates were unavailable, we calculated crude ORs (cORs), 95% CIs, and two-tailed p-values from 2×2 tables using Fisher's exact test. For zero-cell tables, a +0.5 continuity correction (Haldane-Anscombe) was applied.

Prevalence of current ENDS use in defined IBD cohorts was summarized descriptively. Un-pooled forest plots were constructed on a logarithmic scale to visually display study-specific effect estimates. All study-level data handling, exploratory calculations, and figure generation were performed using Python version 3.13 (Python Software Foundation, Beaverton, Oregon, USA), with the libraries pandas and NumPy for data management, SciPy and statsmodels for statistical calculations, and matplotlib for data visualization. A two-sided p-value < 0.05 was considered statistically significant.

Certainty of Evidence

We considered the overall strength and limitations of the evidence during narrative synthesis, with particular attention to study design, risk of bias, inconsistency across studies, indirectness, imprecision, and potential residual confounding, especially related to combustible smoking history and dual use.

Results

Included Studies

Following screening and full-text assessment, four full-text studies met inclusion criteria for the main synthesis [8,9,12,13]. Across the included studies, 1,869 participants with IBD were included, of whom 178 (9.5%) were identified as ENDS or e-cigarette users (Table 1).

**Table 1:** Included study characteristics CD, Crohn’s disease; EHR, electronic health record; ENDSs, electronic nicotine delivery systems; HNB, heat-not-burn tobacco; IBD, inflammatory bowel disease; UC, ulcerative colitis. *The control group included combustible cigarette smokers (70% never, 25% former, 5% current). †Comparator sample sizes are shown only when fixed at the study level; for Chong and Parigi, comparator denominators varied by disease subgroup and/or outcome assessment.

Study	Region	Design/data source	Population/context	ENDS exposure (n; definition; ascertainment)	Comparator(s)	Smoking history/dual use	Outcome window/key outcomes
Chong et al., (2019) [8]	UK	Cross-sectional clinic audit with exploratory follow-up	Consecutive adult IBD outpatients; N=465 (mixed CD/UC)	Current e-cigarette use (n=15 at baseline); self-report	Combustible cigarette smokers†	High confounding risk: ENDS users were largely or entirely ex-smokers; dual use not clearly controlled	Baseline prevalence; selected clinical outcomes in a small follow-up subset (~21 months)
Sheehan et al.,(2023) [9]	US	Retrospective matched case-control (1:2), EHR-based cohort	Adult specialty IBD cohort; N=378; CD and UC strata reported	Current nicotine-containing e-cigarette use (n=127); EHR documentation with chart confirmation	Non-vaping controls (n=251)*	Dual use present; smoking status adjusted and stratified (never vs ever smokers)	Two years after index date; composite outcome (biologic initiation/therapy change, hospitalization, surgery) and component outcomes
Parigi et al., (2025) [13]	Multicentre Europe (Italy, Spain, France; 9 centres)	Retrospective postoperative cohort	Post-ileocolonic resection Crohn’s disease cohort; N=937	Exclusive postoperative e-cigarette use (n=33) between surgery and endoscopy; EHR review with direct confirmation	Nonsmokers/no tobacco; additional combustible cigarette and HNB groups†	Design restriction excluded switching and concurrent multi-product use	Endoscopy at 6–12 months (≤1 year); primary outcome: endoscopic recurrence (Rutgeerts ≥i2); secondary outcomes: calprotectin and imaging activity
Malibary et al., (2021) [12]	Saudi Arabia	Observational UC cohort (flare-focused; cross-sectional/retrospective assessment)	Single-centre adult UC cohort; N=89	Current vaping (n=3); self-report/EHR	Non-vaping UC patients (n=86)	Prior smoking history and dual use not clearly controlled; vaping exposure extremely sparse	Time anchoring unclear; contributes UC flare recurrence comparison

Exposure and Comparator Definitions

Across the four included full-text studies, e-cigarette/vaping exposure was generally defined as current use, with limited reporting of former use. Ascertainment of exposure varied by design, including self-report questionnaire/survey [8,12], electronic health record documentation [9], and medical record review during a postoperative interval [13]. Comparator definitions differed across studies and included combustible cigarette smokers, non-vaping controls, and non-smokers/no tobacco exposure groups. Importantly, none of the included studies used biochemical verification of nicotine/tobacco exposure (e.g., cotinine assays or hair nicotine), which may have increased the risk of exposure misclassification, particularly in the setting of dual use or recent switching.

Smoking History and Dual Use Handling

Smoking history and dual use were handled inconsistently across studies and remain important threats to internal validity.

In the study by Sheehan et al., cases were defined as current nicotine-containing e-cigarette users and included substantial dual use. Traditional cigarette smoking status among cases was 13% never, 50% former, and 37% current smokers, indicating that 37% of ENDS users were concurrent cigarette smokers. Controls were non-vaping controls with no prior vaping history, but controls were not uniformly non-smokers (70% never, 25% former, 5% current smokers). Smoking status was statistically controlled for by multivariable adjustment including smoking status and by pre-specified stratified analyses among never versus ever smokers, with similar null findings across strata [9].

In the study by Chong et al., all e-cigarette users had previous cigarette smoking exposure, and 13 of the current e-cigarette users had stopped smoking completely, implying that a minority remained dual users. The authors explicitly acknowledged that this overlap between current vaping and prior smoking was the principal confounder, and no multivariable adjustment was performed to isolate ENDS effects from smoking history [8].

Malibary et al. reported that vaping exposure was extremely sparse (three current vapers) and comparisons were largely unadjusted. Multivariable logistic regression for recurrent flares did not identify vaping or cigarette smoking as independent predictors, offering limited control for tobacco-related confounding [12].

Finally, the study by Parigi et al. used design restrictions to reduce confounding by smoking behavior changes and dual use, excluding patients who switched products postoperatively and those who used multiple products concurrently. Exclusive e-cigarette use was also verified by direct confirmation. The primary comparison used nonsmokers as reference [13].

Overall, only Sheehan et al. and Parigi et al. meaningfully mitigated smoking-related confounding, through adjustment and stratification or through design restriction and direct verification, respectively [9,13]. The remaining studies carry a high risk of residual confounding given the near-complete overlap between current vaping and prior cigarette exposure together with minimal analytic control.

Risk of Bias

Risk of bias assessments are summarized in the Appendices. Key limitations across studies included likely residual confounding by combustible smoking history, potential exposure misclassification (self-report or incomplete documentation), and imprecision due to small vaping groups in several strata (particularly UC).

Prevalence of E-cigarette/Vaping Use in IBD Cohorts

Three studies contributed prevalence estimates from defined IBD populations. In a UK outpatient IBD audit, current e-cigarette use was reported by 3.2% (15/465) of the clinic cohort [8]. In a Saudi UC cohort, current vaping prevalence was reported by 3.4% (3/89) [12]. In a multicenter postoperative CD cohort, exclusive postoperative e-cigarette use was reported in 3.5% (33/937) [13]. The matched case-control design of Sheehan et al. did not allow estimates of prevalence because exposure groups were defined by design [9].

Clinical Outcomes

CD outcomes: Three studies reported outcomes in patients with CD using e-cigarettes, in two distinct contexts: general CD populations and postoperative recurrence (Table 2) [8,9,13].

**Table 2:** Crohn's disease outcomes by E-cigarette use aOR, adjusted odds ratio; cOR, crude odds ratio; CI, confidence interval; FC, fecal calprotectin; NR, not reported. * Calculated by reviewers from extracted or inferred study data; not explicitly reported in this format in the source manuscript. Event counts for Sheehan 2023 and Parigi 2025 were inferred by rounding published percentages to the nearest whole patient; inferred counts may differ by ±1 because of rounding. Reviewer-calculated cORs and p-values were derived from extracted or inferred 2×2 tables using two-sided Fisher's exact test. [†] For zero-cell tables, cORs and 95% CIs were calculated using a +0.5 continuity correction (Haldane–Anscombe). Adjusted odds ratios and corresponding p-values were extracted directly from the source manuscripts when reported (Sheehan 2023 and the primary Parigi 2025 comparison versus nonsmokers). For Parigi 2025, denominators varied across outcomes according to availability of postoperative assessments (e.g., Rutgeerts score, fecal calprotectin, imaging) at the 6–12-month follow-up.

Study	Outcome	E-cig users	Comparator	Effect estimate (primary)	Supporting stats (secondary)
Chong et al., (2019) [8]	Composite disease-related outcome (general CD; ~21 months)	1/6 (17%)	Cigarette smokers: 28/53 (53%)	cOR 0.18 (95% CI 0.02–1.63)	p=0.19
	Symptoms of active IBD	1/6 (17%)	Cigarette smokers: 27/53 (51%)	cOR 0.19 (95% CI 0.02–1.76)	p=0.20
	Objective inflammation	1/6 (17%)	Cigarette smokers: 26/53 (49%)	cOR 0.21 (95% CI 0.02–1.90)	p=0.20
	Steroid course for IBD	0/6 (0%)	Cigarette smokers: 5/53 (9%)	cOR 0.68 (95% CI 0.03–13.74) †	p=1.00
	Change of maintenance med	0/6 (0%)	Cigarette smokers: 14/53 (26%)	cOR 0.21 (95% CI 0.01–3.96) †	p=0.32
	Need for surgery/hospital	1/6 (17%)	Cigarette smokers: 12/53 (23%)	cOR 0.68 (95% CI 0.07–6.43)	p=1.00
Sheehan et al. (2023) [9]	Composite clinical outcome (general CD; 2 y)	21/80 (26%) *	Non-vaping controls: 54/158 (34%) *	aOR 0.82 (95% CI 0.36–1.87)	p=0.63
	IBD-hospitalization (2 y)	14/80 (18%) *	Non-vaping controls: 33/158 (21%) *	aOR 1.20 (95% CI 0.42–3.42)	p=0.74
	IBD-surgery (2 y)	11/80 (14%) *	Non-vaping controls: 21/158 (13%) *	aOR 1.84 (95% CI 0.56–6.02)	p=0.31
	Steroid use (2 y)	25/80 (31%) *	Non-vaping controls: 52/158 (33%) *	aOR 0.76 (95% CI 0.34–1.68)	p=0.49
	New biologic initiation (2 y)	10/80 (13%) *	Non-vaping controls: 35/158 (22%) *	aOR 0.56 (95% CI 0.21–1.54)	p=0.26
Parigi et al., (2025) [13]	Endoscopic recurrence (Rutgeerts ≥i2; postoperative CD)	19/31 (61%) *	Nonsmokers: 223/547 (40.8%) *	aOR 2.02 (95% CI 0.96–4.39)	p=0.067
			Cigarette smokers: 100/144 (69.4%) *	cOR 0.70 (95% CI 0.31–1.56)	p=0.40 *
	Endoscopic recurrence among prophylaxis recipients	16/25 (64%) *	Nonsmokers: 183/440 (41.6%) *	cOR 2.50 (95% CI 1.08–5.77)	p=0.037 *
			Cigarette smokers: 84/120 (70%) *	cOR 0.76 (95% CI 0.31–1.88)	p=0.64 *
	Biochemical activity (FC ≥150 μg/g)	12/26 (46%) *	Nonsmokers: 70/266 (26.3%) *	cOR 2.40 (95% CI 1.06–5.44)	p=0.039 *
			Cigarette smokers: 62/96 (64.6%) *	cOR 0.47 (95% CI 0.20–1.13)	p=0.114 *
	Transmural activity (imaging)	8/18 (44%) *	Nonsmokers: 80/200 (40.0%) *	cOR 1.20 (95% CI 0.45–3.17)	p=0.80 *
			Cigarette smokers: 41/69 (59.4%) *	cOR 0.55 (95% CI 0.19–1.56)	p=0.294 *
	Mean Rutgeerts score	1.76 (n=33)	Nonsmokers: 1.18 (n=547)	Mean diff 0.58 *	p=0.028
			Cigarette smokers: 2.08 (n=144)	Mean diff -0.32 *	p = NR

Disease activity and complications (general CD): In prospective follow-up from a clinic audit, Chong et al. reported a composite disease-related outcome (defined as symptoms of active IBD, objective inflammation, steroid course for IBD, change of IBD maintenance medication, or need for hospitalization or surgery) over approximately 21 months among a small CD subset (which included just six e-cigarette users). The composite disease-related outcome occurred in 17% (1/6) of e-cigarette users versus 53% (28/53) of combustible cigarette smokers (cOR 0.18; 95% CI 0.02-1.63) [8].

Similarly, Sheehan et al. found no difference in a composite clinical outcome over two years (defined as new biologic initiation and/or therapy change, hospitalization, or surgery). Their composite clinical outcome occurred in 26% of e-cigarette users versus 34% of non-vaping controls (aOR 0.82; 95% CI 0.36-1.87) [9].

Postoperative recurrence (post-ileocolonic resection): In a multicenter postoperative CD cohort, Parigi et al. reported higher endoscopic recurrence among postoperative e-cigarette users compared with non-smokers. At one year, endoscopic recurrence (Rutgeerts ≥i2) occurred in 60.6% of exclusive e-cigarette users and 40.8% of nonsmokers (unadjusted p = 0.038). In multivariable regression (reference: nonsmokers), the association for e-cigarettes showed a similar but non-significant trend (aOR 2.02; 95% CI 0.96-4.39; p = 0.067) [13].

Biochemical and transmural activity (postoperative): Parigi et al. also examined objective postoperative activity proxies around the time of endoscopy. Biochemical disease activity (defined as fecal calprotectin ≥150 μg/g) was more frequent in e-cigarette users (46.2%) than in nonsmokers (26.3%; p = 0.039; cOR 2.40; 95% CI 1.06-5.44). Mean fecal calprotectin values were 185 μg/g in e-cigarette users versus 120 μg/g in nonsmokers (versus 300 μg/g in combustible cigarette smokers). Transmural activity on ultrasound did not differ significantly between e-cigarette users (44.4%) and nonsmokers (40.0%; cOR 1.20; 95% CI 0.45-3.17) [13].

Effect of prophylactic therapy (postoperative): Within the postoperative cohort, Parigi et al. evaluated the impact of smoking habits on patients actively receiving postoperative prophylaxis (e.g., biologics or azathioprine initiated within two months of surgery). Among this subset, endoscopic recurrence was more frequent in exclusive e-cigarette users than in nonsmokers (63% vs 41.6%; cOR 2.50; 95% CI 1.08-5.77; p = 0.037) [13].

**Figure 2:**
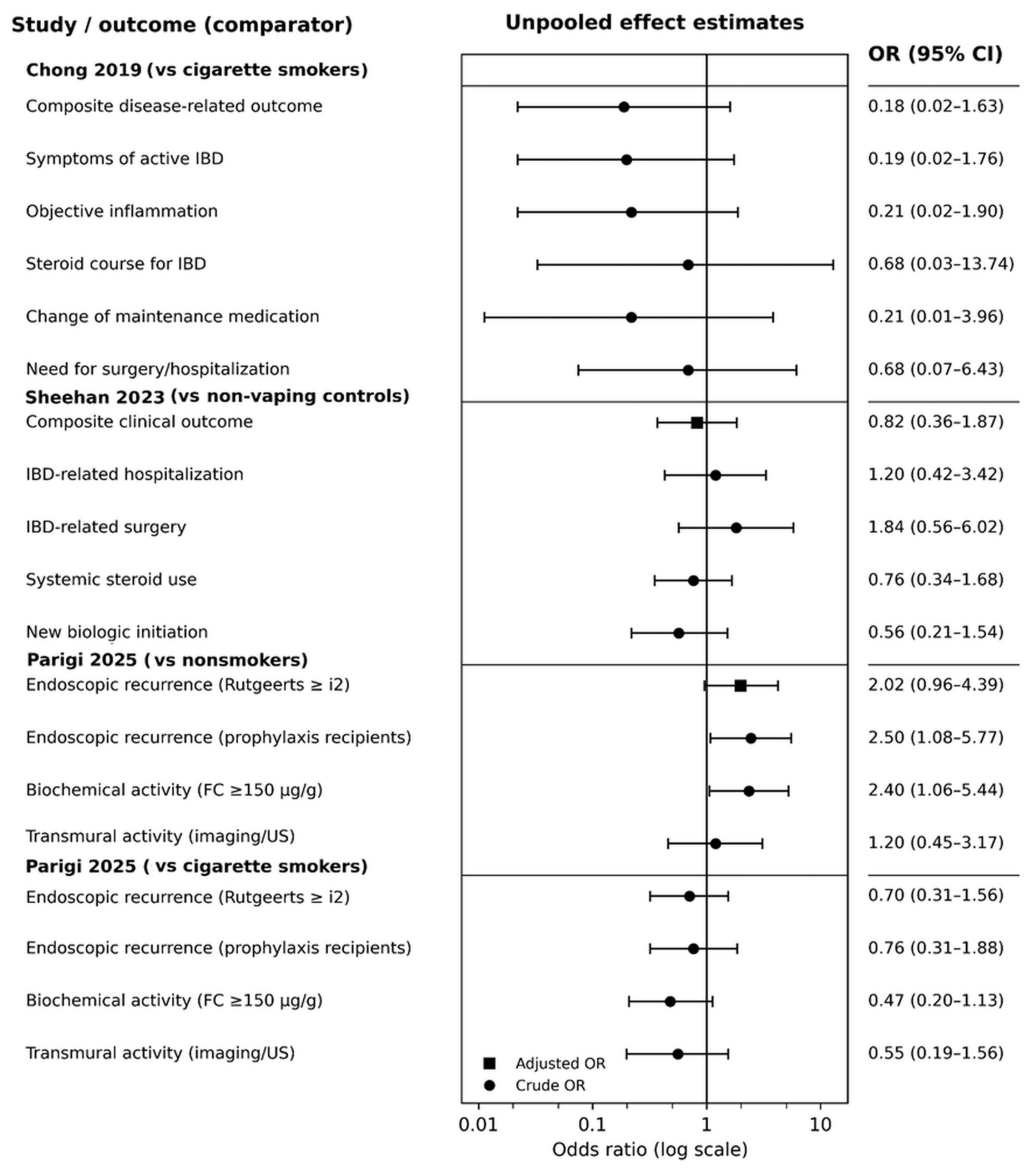
Unpooled forest plot of Crohn’s disease outcomes associated with ENDS/e-cigarette use Unpooled forest plot of Crohn’s disease outcomes associated with ENDS/e-cigarette use. Points show odds ratios with 95% confidence intervals; squares denote adjusted estimates and circles crude estimates. Estimates are grouped by study, comparator, and clinical context. No pooled estimate is shown because of clinical and methodological heterogeneity. The x-axis is logarithmic. FC, fecal calprotectin; US, ultrasound.

These study-specific CD estimates are displayed in an unpooled forest plot (Figure 2).

Ulcerative Colitis Outcomes

Three studies contributed UC-relevant outcome data, but exposed sample sizes were small in two studies (Table 3) [8,9,12].

**Table 3:** Ulcerative colitis outcomes by E-cigarette use aOR, adjusted odds ratio; cOR, crude odds ratio; CI, confidence interval; NR, not reported. * Calculated by reviewers from extracted or inferred study data; not explicitly reported in this format in the source manuscript. Event counts for Sheehan 2023 were inferred by rounding published percentages to the nearest whole patient; inferred counts may differ by ±1 because of rounding. Reviewer-calculated cORs and p-values were derived from extracted or inferred 2×2 tables using a two-sided Fisher exact test. [†] For zero-cell tables, cORs and 95% CIs were calculated using a +0.5 continuity correction (Haldane–Anscombe). For Sheehan 2023, the adjusted odds ratio for the overall composite outcome and the study-reported p-values for individual ulcerative colitis outcomes were extracted directly from the source manuscript. Study-reported p-values for individual outcomes are adjusted multivariable values and are not directly paired with the reviewer-calculated cORs shown in this table.

Study	Outcome	E-cig users	Comparator	Effect estimate (primary)	Supporting stats (secondary)
Malibary et al., (2021) [12]	Recurrent flares	3/3 (100%)	Non-users: 43/86 (50%)	cOR 7.00 (95% CI 0.35–139.60) †	p=0.09
			Cigarette smokers: 3/8 (37.5%)	cOR 11.0 (95% CI 0.43–284.32) †	p=0.18 *
Chong et al., (2019) [8]	Composite disease-related outcome	3/5 (60%)	Cigarette smokers: 4/16 (25%)	cOR 4.50 (95% CI 0.54–37.38)	p=0.28 *
	Symptoms of active IBD	3/5 (60%)	Cigarette smokers: 4/16 (25%)	cOR 4.50 (95% CI 0.54–37.38)	p=0.28 *
	Objective inflammation	2/5 (40%)	Cigarette smokers: 3/16 (19%)	cOR 2.89 (95% CI 0.32–25.68)	p=0.55 *
	Steroid course for IBD	3/5 (60%)	Cigarette smokers: 3/16 (19%)	cOR 6.50 (95% CI 0.73–57.77)	p=0.12 *
	Change of maintenance medication	3/5 (60%)	Cigarette smokers: 1/16 (6%)	cOR 22.50 (95% CI 1.51–335.53)	p=0.028 *
	Need for surgery or hospitalization	0/5 (0%)	Cigarette smokers: 2/16 (12%)	cOR 0.64 (95% CI 0.03–16.03) †	p=1.00 *
Sheehan et al., (2023) [9]	Composite clinical outcome (2 y)	13/47 (28%) *	Non-vaping controls: 22/93 (24%) *	aOR 1.05 (95% CI 0.33–3.39)	p=NR
	Hospitalization (2 y)	10/47 (21%) *	Non-vaping controls: 15/93 (16%) *	cOR 1.41 (95% CI 0.58–3.42) *	p=0.79
	Surgery (2 y)	6/47 (13%) *	Non-vaping controls: 9/93 (10%) *	cOR 1.37 (95% CI 0.46–4.10) *	p=0.80
	Steroid use (2 y)	16/47 (34%) *	Non-vaping controls: 37/93 (40%) *	cOR 0.78 (95% CI 0.38–1.62) *	p=0.62
	New biologic initiation (2 y)	8/47 (17%) *	Non-vaping controls: 13/93 (14%) *	cOR 1.26 (95% CI 0.48–3.30) *	p=0.50

Disease flares and activity: In the study by Malibary et al., all three e-cigarette users experienced recurrent flares (100%) compared with 43/86 (50%) of non-vapers (p = 0.09; exploratory cOR 7.00; 95% CI 0.35-139.60, with +0.5 continuity correction). Recurrent flares were also more frequent among e-cigarette users than among cigarette smokers (3/3 vs 3/8; 37.5%; exploratory cOR 11.0; 95% CI 0.43-284.32; p = 0.18). Both comparisons are extremely imprecise because of the very small exposed group [12].

In the study by Chong et al. (clinic audit follow-up subset), the composite disease-related outcome (defined as symptoms of active IBD, objective inflammation, steroid course for IBD, change of IBD maintenance medication, or need for hospitalization or surgery) occurred in 3/5 (60%) UC e-cigarette users versus 4/16 (25%) UC combustible cigarette smokers (cOR 4.50; 95% CI 0.54-37.38) [8].

Composite clinical outcomes: In UC patients from the study by Sheehan et al., the composite outcome (new biologic initiation, change of existing biologic therapy, IBD-related hospitalization, or surgery) occurred in 13/47 (28%) e-cigarette users versus 22/93 (24%) non-vaping controls (aOR 1.05; 95% CI 0.33-3.39). For UC component outcomes (hospitalization, surgery, steroid use, start of a new biologic), differences were small and crude odds ratios all had 95% CIs crossing one [9].

**Figure 3:**
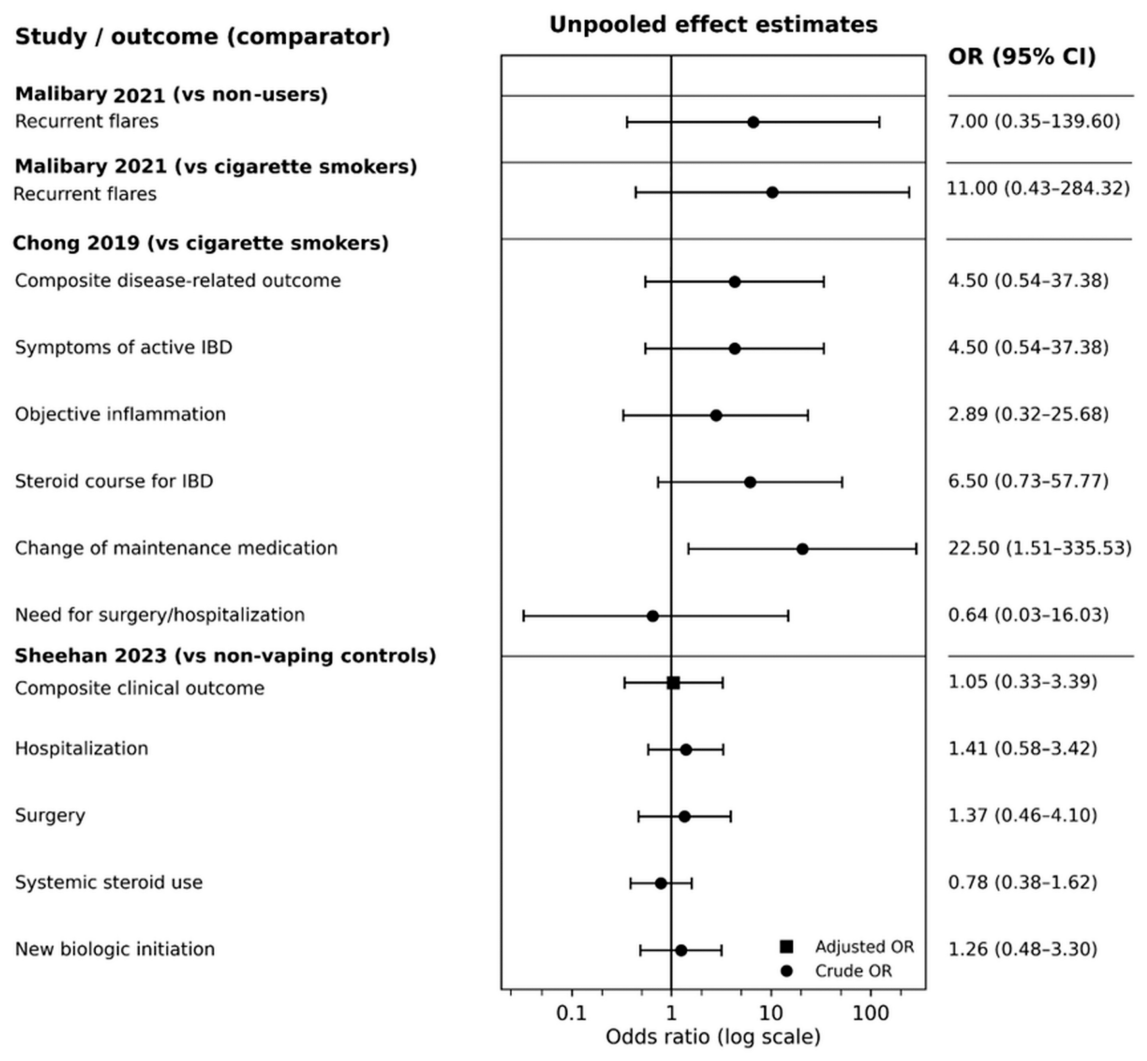
Unpooled forest plot of ulcerative colitis outcomes associated with ENDS/e-cigarette use Unpooled forest plot of ulcerative colitis outcomes associated with ENDS/e-cigarette use. Points show odds ratios with 95% confidence intervals; squares denote adjusted estimates and circles crude estimates. Estimates are grouped by study and comparator. No pooled estimate is shown because of heterogeneity and sparse exposed sample sizes. The x-axis is logarithmic.

These study-specific UC estimates are displayed in an unpooled forest plot (Figure 3).

Comparative Context: E-cigarettes Relative to Combustible Cigarettes

Three studies provided compared outcomes among nicotine exposure groups [8,12,13]. In the postoperative CD cohort, endoscopic recurrence (defined as Rutgeerts ≥i2) occurred in 69.4% of combustible cigarette smokers, 63.9% of heat-not-burn tobacco users, 60.6% of exclusive e-cigarette users, and 40.8% of nonsmokers. In multivariable analysis (reference: nonsmokers), the adjusted odds of recurrence were higher for combustible cigarettes (aOR 3.49; 95% CI 2.34-5.28; p < 0.001) and heat-not-burn tobacco (aOR 2.76; 95% CI 1.36-5.82; p = 0.006), while the association for e-cigarettes was directionally elevated but not statistically significant (aOR 2.02; 95% CI 0.96-4.39; p = 0.067) [13].

In the UK clinic audit exploratory follow-up, complication rates were not significantly different between e-cigarette users and cigarette smokers in CD (17% [1/6] vs 53% [28/53]; cOR 0.18; 95% CI 0.02-1.63; p = 0.19) or UC (60% [3/5] vs 25% [4/16]; cOR 4.50; 95% CI 0.54-37.38; p = 0.28), with both comparisons limited by very small e-cigarette denominators [8].

In the study by Malibary et al., recurrent flares in UC were more frequent in e-cigarette users than in cigarette smokers (100% [3/3] vs 37.5% [3/8]), but the estimate was extremely imprecise (cOR 11.0; 95% CI 0.43-284.32; p = 0.18) because of the very small number of exposed patients [12].

Quantitative Synthesis (Meta-Analysis)

Quantitative pooling was not performed because of limited clinical and methodological comparability across studies, including differences in comparator frameworks (smokers versus non-vapers versus non-smokers), outcome definitions, follow-up windows, and the distinct postoperative recurrence context, which would make pooled estimates difficult to interpret and at risk of being misleading.

Discussion

In this systematic review evaluating ENDS/e-cigarette exposure in adults with IBD, the most methodologically informative evidence for general IBD outcomes came from a matched case-control study which did not demonstrate higher odds of clinically consequential outcomes, including hospitalization, surgery, or treatment escalation, among e-cigarette users compared with non-vaping controls [9]. In a high-risk postoperative CD cohort, the adjusted association between exclusive e-cigarette use and endoscopic recurrence versus nonsmoking did not reach conventional statistical significance, although crude recurrence rates and secondary objective inflammatory markers were higher among e-cigarette users [13]. Taken together, the available evidence does not show a consistent, reproducible signal across settings. Findings appear to vary by clinical context, outcome type, and the extent to which smoking history and dual use were measured and addressed. Overall certainty of evidence is low to very low because of residual confounding and imprecision.

Our findings should be interpreted against the well-established literature that combustible cigarette smoking worsens CD course and increases the risk of relapse and surgery [3,14,15]. In UC, smoking is inversely associated with UC onset and, less consistently, with some measures of severity [6,16]. This established divergence by IBD phenotype provides a clinically coherent explanation for why small observational datasets may show seemingly discordant directions when e-cigarette users are compared with smokers in UC (where smoking may appear protective by certain proxies), while in CD, particularly in the postoperative setting, any nicotine/tobacco exposure may track with higher recurrence risk.

A key interpretive challenge across the ENDS-IBD literature is that e-cigarette use in IBD often occurs in the context of prior or concurrent combustible cigarette exposure. The largest general outcomes study emphasized analytic handling of smoking status but not biological objective confirmation and found no association between current e-cigarette use and the composite outcome after adjustment [9]. Notably, in the study by Sheehan et al., current combustible cigarette smoking was more common among e-cigarette users than among non-vaping controls (37% vs 5%), which would be expected to bias unadjusted comparisons toward worse outcomes in the e-cigarette group. Despite this imbalance, adjusted analyses did not show higher odds of the composite outcome [9]. The clinic audit similarly highlighted that vaping frequently co-occurred with a conventional smoking history, and its exploratory follow-up analyses were underpowered [8]. In the UC flare cohort, while the unadjusted rate of flares in vapers was 100%, the extremely small exposed sample size (n = 3) precludes reliable inference [12]. Furthermore, their multivariable logistic regression found that only a family history of UC (OR 5.3) and fecal incontinence (OR 4.7) were independent predictors of recurrent flares, failing to identify e-cigarettes as a significant risk factor for flares [12]. In the postoperative cohort, the exposure contrast (exclusive e-cigarette use versus nonsmoking) is clinically meaningful, but residual confounding by baseline postoperative risk factors and prior smoking intensity remains plausible even when multivariable adjustment is applied [13].

Mechanistically, ENDS exposure is not equivalent to nicotine exposure alone. Although nicotine has been investigated as a potential modulator of colitis biology including historical trials of transdermal nicotine in UC [17], ENDS aerosols also contain solvents and thermal degradation products that may influence epithelial permeability and inflammatory signaling beyond nicotine mediated pathways [11]. This distinction limits the extent to which UC nicotine responsiveness can be extrapolated to modern vaping products, particularly given variability in device temperature, constituents, and intensity of use.

Postoperative CD recurrence is a biologically and clinically distinct context, characterized by recurrent endoscopic lesions at the ileocolonic anastomosis and/or neoterminal ileum and typically assessed using the Rutgeerts score or its modifications [18,19]. Smoking is a major, consistently replicated risk factor for postoperative recurrence, and cessation is emphasized in recurrence prevention strategies [15,20]. The postoperative cohort in this review extends that risk-stratification discussion to newer nicotine delivery products, showing a gradient where nonsmokers experienced the lowest recurrence and combustible cigarette smokers the highest, with e-cigarettes and heat-not-burn products in between [13].

The apparent discrepancy in e-cigarette outcomes among the included studies may be largely explained by the variation in chosen endpoints. Sheehan et al. utilized a composite clinical outcome consisting of hospitalization, surgery, and biologic initiation or switch [9]. While clinically significant, these milestones are partially subjective and can be heavily influenced by physician prescribing behaviors, healthcare access, and patient reported symptoms. In contrast, Parigi et al. relied on objective subclinical measures of disease activity [13]. Beyond demonstrating higher rates of macroscopic endoscopic recurrence (Rutgeerts score ≥2), Parigi et al. highlighted a significant increase in objective markers of inflammation, including elevated fecal calprotectin (≥150 μg/g) in 46.2% compared with only 26.3% of nonsmokers [13]. Taken together, these findings suggest that ENDS exposure may be associated with objective mucosal and biochemical inflammation, even if this underlying damage has not yet translated into subjective clinical events or hospitalizations within a short two-year follow-up window. These findings are consistent with higher objective inflammatory activity among postoperative exclusive e-cigarette users compared with nonsmokers, but study design, residual confounding, and exposure-history carryover remain plausible explanations; therefore, results should be interpreted as hypothesis-generating rather than causal.

Building on this evidence of disease activity, Parigi et al. also suggest that recurrence remained high among e-cigarette users despite postoperative prophylaxis, with recurrence rates of 63.0% versus 41.6% in nonsmokers [13]. This may indicate that standard prophylaxis does not fully offset recurrence risk in patients who continue ENDS use, although causal inference remains limited.

Clinical Implications

The evidence base is insufficient to conclude that ENDS use is safe with respect to IBD activity or postoperative recurrence, particularly in CD. At the same time, the current data do not consistently demonstrate that e-cigarette use worsens short- to intermediate-term general IBD outcomes compared with non-vaping controls in nonoperative settings. This leaves clinicians operating in a harm-reduction grey zone. Many patients consider ENDS as a bridge away from combustible smoking, yet dual use is common and may diminish any potential risk reduction.

Smoking cessation literature in the general population suggests nicotine-containing e-cigarettes can improve quit rates compared with some comparators [21], but current recommendations remain jurisdiction dependent. In the United Kingdom, the National Institute for Health and Care Excellence (NICE) and the National Health Service (NHS) provide guidance that is relatively more supportive of nicotine vaping as a harm reduction or cessation option for adults who smoke, although such products are generally not licensed as medicines [22]. By contrast, the United States Preventive Services Task Force (USPSTF) states that evidence is insufficient to support e-cigarettes for smoking cessation and recommends established cessation interventions with proven effectiveness and safety instead [23], and the European Respiratory Society does not endorse e-cigarettes as a population level harm reduction or smoking cessation strategy [24].

Accordingly, in IBD practice, a pragmatic approach is to (1) prioritize complete cessation of combustible cigarettes given their established harms in CD and postoperative recurrence; (2) explicitly assess dual use and prior smoking intensity; and (3) preferentially use evidence-based cessation pharmacotherapies and behavioral support while acknowledging that some patients will use ENDS as part of a cessation trajectory. If patients choose ENDSs during a smoking-cessation attempt, clinicians should advise complete cessation of combustible tobacco and, ultimately, cessation of ENDSs once relapse risk is low [22].

Strengths and Limitations

This review applied PRISMA-aligned methods and used a prespecified analytic approach that separated postoperative CD recurrence from general clinical outcomes, avoiding inappropriate mixing. We synthesized findings by disease subtype (CD versus UC) and by clinical context (postoperative versus nonoperative), reflecting differences in endpoint definition and underlying risk. We also summarized prevalence of ENDS use in defined IBD populations and assessed risk of bias with design-appropriate tools.

However, major limitations constrain inference. First, residual confounding by combustible cigarette smoking history and dual use is likely and may operate in either direction, limiting interpretability. Most evidence is observational and therefore vulnerable to residual confounding, especially by smoking history, baseline disease severity, and treatment intensity. Exposure ascertainment is often self reported or incompletely documented, with sparse granularity on device type, nicotine concentration, intensity/dose, and transitions over time. Small exposed sample sizes (particularly for UC) yield marked imprecision and unstable point estimates. Finally, heterogeneity in comparator frameworks (non-vapers versus smokers versus nonsmokers), outcome constructions, and follow-up windows limits cross-study comparability and makes pooling inappropriate.

Research Implications

The immediate research priority is more rigorous study of this exposure in well-defined clinical cohorts with standardized exposure definitions, objective biological verification where feasible, and longitudinal capture of transitions (exclusive vaping, exclusive smoking, dual use, cessation). Future studies should explicitly stratify by (or emulate) clinically meaningful counterfactuals: vaping as a cessation aid among current smokers versus vaping initiation among never-smokers, with careful adjustment for baseline severity and healthcare utilization. Postoperative CD deserves targeted investigation because objective endoscopic endpoints reduce outcome misclassification and align closely with clinically actionable decisions. Finally, consensus-driven development of a core outcome set for ENDS-IBD studies would materially improve comparability and enable future meta-analysis.

## Conclusions

This systematic review summarizes the emerging clinical evidence on ENDS use in adults with IBD. In nonoperative settings, available evidence does not show a consistent association between current e-cigarette use and major clinical outcomes such as treatment escalation, hospitalization, or surgery. In postoperative CD, the available data suggest a possible signal for higher endoscopic recurrence among exclusive e-cigarette users compared with nonsmokers, but the evidence remains insufficient for causal inference.

Overall certainty is low to very low because of observational study designs, small exposed groups, residual confounding from prior or concurrent combustible cigarette use, and heterogeneous outcome definitions. Clinicians should not assume that e-cigarette use is risk-free in IBD, particularly after CD surgery. Larger prospective studies with standardized exposure definitions, objective exposure verification, and objective disease-activity outcomes are needed.

## Appendices

**Table 4:** PRISMA 2020 checklist PRISMA: Preferred Reporting Items for Systematic Reviews and Meta-Analyses

Section and Topic	Item #	Checklist item	Location where the item is reported
TITLE			
Title	1	Identify the report as a systematic review.	Title & Abstract
ABSTRACT			
Abstract	2	See the PRISMA 2020 for Abstracts checklist.	Abstract
INTRODUCTION			
Rationale	3	Describe the rationale for the review in the context of existing knowledge.	Introduction
Objectives	4	Provide an explicit statement of the objective(s) or question(s) the review addresses.	Introduction (final paragraph)
METHODS			
Eligibility criteria	5	Specify the inclusion and exclusion criteria for the review and how studies were grouped for the syntheses.	Methods: Eligibility Criteria; Data synthesis
Information sources	6	Specify all databases, registers, websites, organisations, reference lists and other sources searched or consulted to identify studies. Specify the date when each source was last searched or consulted.	Methods: Literature Search
Search strategy	7	Present the full search strategies for all databases, registers and websites, including any filters and limits used.	Methods: Literature Search; Supplemental material 2
Selection process	8	Specify the methods used to decide whether a study met the inclusion criteria of the review, including how many reviewers screened each record and each report retrieved, whether they worked independently, and if applicable, details of automation tools used in the process.	Methods: Study selection
Data collection process	9	Specify the methods used to collect data from reports, including how many reviewers collected data from each report, whether they worked independently, any processes for obtaining or confirming data from study investigators, and if applicable, details of automation tools used in the process.	Methods: Data extraction
Data items	10a	List and define all outcomes for which data were sought. Specify whether all results that were compatible with each outcome domain in each study were sought (e.g. for all measures, time points, analyses), and if not, the methods used to decide which results to collect.	Methods: Eligibility Criteria; Data extraction
	10b	List and define all other variables for which data were sought (e.g. participant and intervention characteristics, funding sources). Describe any assumptions made about any missing or unclear information.	Methods: Data extraction
Study risk of bias assessment	11	Specify the methods used to assess risk of bias in the included studies, including details of the tool(s) used, how many reviewers assessed each study and whether they worked independently, and if applicable, details of automation tools used in the process.	Methods: Risk of bias assessment
Effect measures	12	Specify for each outcome the effect measure(s) (e.g. risk ratio, mean difference) used in the synthesis or presentation of results.	Methods: Data synthesis
Synthesis methods	13a	Describe the processes used to decide which studies were eligible for each synthesis (e.g. tabulating the study intervention characteristics and comparing against the planned groups for each synthesis (item #5)).	Methods: Eligibility Criteria, Data synthesis
	13b	Describe any methods required to prepare the data for presentation or synthesis, such as handling of missing summary statistics, or data conversions.	Methods: Data extraction; Data synthesis
	13c	Describe any methods used to tabulate or visually display results of individual studies and syntheses.	Methods: Data synthesis; Tables 1–3; Figures 1–3
	13d	Describe any methods used to synthesize results and provide a rationale for the choice(s). If meta-analysis was performed, describe the model(s), method(s) to identify the presence and extent of statistical heterogeneity, and software package(s) used.	Methods: Data synthesis; Results: Quantitative synthesis
	13e	Describe any methods used to explore possible causes of heterogeneity among study results (e.g. subgroup analysis, meta-regression).	Methods: Data synthesis
	13f	Describe any sensitivity analyses conducted to assess robustness of the synthesized results.	Not reported
Reporting bias assessment	14	Describe any methods used to assess risk of bias due to missing results in a synthesis (arising from reporting biases).	Supplemental material 3
Certainty assessment	15	Describe any methods used to assess certainty (or confidence) in the body of evidence for an outcome.	Methods: Certainty of evidence
RESULTS			
Study selection	16a	Describe the results of the search and selection process, from the number of records identified in the search to the number of studies included in the review, ideally using a flow diagram.	Results: Study selection, Figure 1
	16b	Cite studies that might appear to meet the inclusion criteria, but which were excluded, and explain why they were excluded.	Not reported (reasons summarised in Figure 1, but excluded studies are not individually cited)
Study characteristics	17	Cite each included study and present its characteristics.	Results: Included study characteristics / Exposure and comparator definitions / Smoking history and dual use handling; Table 1
Risk of bias in studies	18	Present assessments of risk of bias for each included study.	Results: Risk of bias; Supplementary Table 3
Results of individual studies	19	For all outcomes, present, for each study: (a) summary statistics for each group (where appropriate) and (b) an effect estimate and its precision (e.g. confidence/credible interval), ideally using structured tables or plots.	Results: Clinical outcomes; Tables 2–3; Figures 2–3
Results of syntheses	20a	For each synthesis, briefly summarise the characteristics and risk of bias among contributing studies.	Results: Exposure and comparator definitions / Smoking history and dual use handling / Risk of bias
	20b	Present results of all statistical syntheses conducted. If meta-analysis was done, present for each the summary estimate and its precision (e.g. confidence/credible interval) and measures of statistical heterogeneity. If comparing groups, describe the direction of the effect.	Results: Clinical outcomes; Results: Quantitative synthesis
	20c	Present results of all investigations of possible causes of heterogeneity among study results.	Methods: Data synthesis. Results: Smoking history and dual use handling
				20d	Present results of all sensitivity analyses conducted to assess the robustness of the synthesized results.	Not reported
				Reporting biases	21	Present assessments of risk of bias due to missing results (arising from reporting biases) for each synthesis assessed.	Supplemental material 3
	Certainty of evidence	22	Present assessments of certainty (or confidence) in the body of evidence for each outcome assessed.	Abstract; Discussion; Conclusion
DISCUSSION			
Discussion	23a	Provide a general interpretation of the results in the context of other evidence.	Discussion
	23b	Discuss any limitations of the evidence included in the review.	Discussion
	23c	Discuss any limitations of the review processes used.	Discussion: Strengths and limitations
	23d	Discuss implications of the results for practice, policy, and future research.	Discussion: Clinical implications; Research implications
OTHER INFORMATION			
Registration and protocol	24a	Provide registration information for the review, including register name and registration number, or state that the review was not registered.	Methods: Protocol and reporting,
	24b	Indicate where the review protocol can be accessed, or state that a protocol was not prepared.	Methods: Protocol and reporting, (PROSPERO registration number provided)
	24c	Describe and explain any amendments to information provided at registration or in the protocol.	Not reported
Support	25	Describe sources of financial or non-financial support for the review, and the role of the funders or sponsors in the review.	Funding
Competing interests	26	Declare any competing interests of review authors.	Conflicts of interest statement
Availability of data, code and other materials	27	Report which of the following are publicly available and where they can be found: template data collection forms; data extracted from included studies; data used for all analyses; analytic code; any other materials used in the review.	Data Availability

**Table 5:** Search strings

Embase
('inflammatory bowel disease'/exp OR 'inflammatory bowel disease*':ti,ab OR IBD:ti,ab OR 'crohn disease'/exp OR 'crohn disease':ti,ab OR 'crohn`s disease':ti,ab OR 'crohns disease':ti,ab OR 'enteritis regionalis':ti,ab OR 'morbus crohn':ti,ab OR 'regional enteritis':ti,ab OR 'regional enterocolitis':ti,ab OR ileocolitis:ti,ab OR 'ulcerative colitis'/exp OR 'chronic ulcerative colitis':ti,ab OR 'colitis ulcerativa':ti,ab OR 'colitis ulcerosa':ti,ab OR 'colitis ulcerosa chronica':ti,ab OR 'histiocytic ulcerative colitis':ti,ab OR 'mucosal colitis':ti,ab OR 'ulcerative colitis':ti,ab OR 'ulcerative colorectitis':ti,ab OR 'ulcerative procto colitis':ti,ab OR 'ulcerative proctocolitis':ti,ab OR 'ulcerous colitis':ti,ab OR ‘colitis gravis’:ti,ab) AND ('electronic cigarette'/exp OR 'e cigarette':ti,ab OR 'e cigarettes':ti,ab OR 'electronic cigarette':ti,ab OR 'electronic cigarettes':ti,ab OR 'electronic nicotine delivery system*':ti,ab OR E-Cig*:ti,ab OR 'vaping'/exp OR 'e-cigarette smoking':ti,ab OR 'electronic cigarette smoking':ti,ab OR 'vaping':ti,ab) AND ('relapse'/exp OR 'relapse':ti,ab OR recurrence*:ti,ab OR 'hospitalization'/exp OR 'hospital stay':ti,ab OR 'hospitalization':ti,ab OR 'short stay hospitalization':ti,ab OR 'corticosteroid'/exp OR 'adrenal cortex hormone':ti,ab OR 'adrenal cortex hormones':ti,ab OR 'adrenal cortical hormone':ti,ab OR 'adrenal cortical hormones':ti,ab OR 'adrenal cortical steroid':ti,ab OR 'adrenal steroid':ti,ab OR 'adrenal steroid hormone':ti,ab OR 'adreno cortical steroid':ti,ab OR 'adreno corticosteroid':ti,ab OR 'adrenocortical hormone':ti,ab OR 'adrenocortical steroid':ti,ab OR 'adrenocorticosteroid':ti,ab OR 'cortical steroid':ti,ab OR 'cortico steroid':ti,ab OR 'corticoid':ti,ab OR 'corticosteroid':ti,ab OR 'corticosteroid agent':ti,ab OR 'corticosteroid calcium':ti,ab OR 'corticosteroid hormone':ti,ab OR 'corticosteroids':ti,ab OR 'surgery'/exp OR 'operation':ti,ab OR 'operative intervention':ti,ab OR 'operative surgery':ti,ab OR 'operative surgical procedure':ti,ab OR 'operative surgical procedures':ti,ab OR 'operative treatment':ti,ab OR 'surgery':ti,ab OR 'surgical care':ti,ab OR 'surgical correction':ti,ab OR 'surgical intervention':ti,ab OR 'surgical management':ti,ab OR 'surgical operation':ti,ab OR 'surgical repair':ti,ab OR 'surgical restoration':ti,ab OR 'surgical therapy':ti,ab OR 'surgical treatment':ti,ab OR 'adverse event'/exp OR 'adverse effect':ti,ab OR 'adverse effects':ti,ab OR 'adverse event*':ti,ab OR 'adverse reaction*':ti,ab OR 'c reactive protein'/exp OR 'c reactive protein':ti,ab OR 'c reaction protein':ti,ab OR 'c-reactive protein':ti,ab OR 'creactive protein':ti,ab OR 'crp':ti,ab OR 'serum c reactive protein':ti,ab OR 'fecal calprotectin'/exp OR ‘fecal calprotectin’:ti,ab)
PubMed
("Inflammatory Bowel Diseases"[Mesh] OR “inflammatory bowel disease*”[tiab] OR IBD[tiab] OR "Crohn Disease"[Mesh] OR “crohn* disease”[tiab] OR “enteritis regionalis”[tiab] OR “morbus crohn”[tiab] OR “regional enteritis”[tiab] OR “regional enterocolitis”[tiab] OR ileocolitis[tiab] OR "Colitis, Ulcerative"[Mesh] OR “ulcerative colitis”[tiab] OR “chronic ulcerative colitis”[tiab] OR “colitis ulcerative”[tiab] OR “colitis ulcerosa”[tiab] OR “colitis ulcerosa chronica”[tiab] OR “histiocytic ulcerative colitis”[tiab] OR “mucosal colitis”[tiab] OR “ulcerative colorectitis”[tiab] OR “ulcerative procto colitis”[tiab] OR “ulcerative proctocolitis”[tiab] OR “ulcerous colitis”[tiab] OR “colitis gravis”[tiab]) AND ("Electronic Nicotine Delivery Systems"[Mesh] OR “electronic nicotine delivery system”[tiab] OR “E-Cigarette*”[tiab] OR E-Cig*[tiab] OR "Vaping"[Mesh] OR vaping*[tiab] OR “e-cigarette smoking”[tiab] OR “electronic cigarette smoking”[tiab]) AND ("Recurrence"[Mesh] OR recurrence*[tiab] OR relapse*[tiab] OR "Hospitalization"[Mesh] OR hospitalization*[tiab] OR “hospital stay*”[tiab] OR "Adrenal Cortex Hormones"[Mesh] OR corticosteroid*[tiab] OR “adrenal cortex hormone*”[tiab] OR “adrenal cortical hormone*”[tiab] OR “adrenal cortical steroid*”[tiab] OR “adrenal steroid*”[tiab] OR “adrenal steroid hormone*”[tiab] OR “adreno cortical steroid*”[tiab] OR “adreno corticosteroid*”[tiab] OR “adrenocortical hormone*”[tiab] OR “adrenocortical steroid*”[tiab] OR adrenocorticosteroid*[tiab] OR “cortical steroid*”[tiab] OR “cortico steroid*”[tiab] OR corticoid*[tiab] OR corticosteroid*[tiab] OR “corticosteroid agent”[tiab] OR “corticosteroid calcium”[tiab] OR “corticosteroid hormone*”[tiab] OR "Surgical Procedures, Operative"[Mesh] OR surger*[tiab] OR operation*[tiab] OR “operative intervention”[tiab] OR “operative surgery”[tiab] OR “operative surgical procedure*”[tiab] OR “operative treatment”[tiab] OR “surgical care”[tiab] OR “surgical correction”[tiab] OR “surgical intervention”[tiab] OR “surgical management”[tiab] OR “surgical operation”[tiab] OR “surgical repair”[tiab] OR “surgical restoration”[tiab] OR “surgical therapy”[tiab] OR “surgical treatment”[tiab] OR “adverse event*”[tiab] OR “adverse reaction*”[tiab] OR "C-Reactive Protein"[Mesh] OR “C-Reactive Protein”[tiab] OR “creactive protein”[tiab] OR crp[tiab] OR “serum c reactive protein”[tiab] OR “fecal calprotectin”[tiab])
Scopus
(INDEXTERMS (“inflammatory bowel disease” OR “crohn disease” OR “ulcerative colitis” ) OR (TITLE-ABS-KEY ("inflammatory bowel disease*" OR "crohn disease" OR "crohn’s disease" OR "enteritis regionalis" OR "morbus crohn" OR "regional enteritis" OR "regional enterocolitis" OR ileocolitis OR "ulcerative colitis" OR "chronic ulcerative colitis" OR "colitis ulcerosa" OR "colitis ulcerosa chronica" OR "histiocytic ulcerative colitis" OR "mucosal colitis" OR "ulcerative colorectitis" OR "ulcerative procto colitis" OR "ulcerative proctocolitis" OR "ulcerous colitis" OR "colitis gravis"))) AND (INDEXTERMS ("Electronic Nicotine Delivery Systems" OR “electronic cigarette”) OR (TITLE-ABS-KEY ("electronic nicotine delivery system*" OR E-Cigarette* OR E-Cig* OR vaping OR "e-cigarette smoking" OR "electronic cigarette smoking"))) AND (INDEXTERMS (relapse OR hospitalization OR “adrenal cortex hormones” OR surgery OR “adverse event” OR “C-reactive protein”) OR (TITLE-ABS-KEY (recurrence* OR relapse* OR hospitalization* OR "hospital stay*" OR corticosteroid* OR "adrenal cortex hormone*" OR "adrenal cortical hormone*" OR "adrenal cortical steroid*" OR "adrenal steroid*" OR "adrenal steroid hormone*" OR "adreno cortical steroid*" OR "adreno corticosteroid*" OR "adrenocortical hormone*" OR "adrenocortical steroid*" OR adrenocorticosteroid* OR "cortical steroid*" OR "cortico steroid*" OR corticoid* OR corticosteroid* OR "corticosteroid agent*" OR "corticosteroid calcium" OR "corticosteroid hormone*" OR surger* OR operation* OR "operative intervention*" OR "operative surgery" OR "operative surgical procedure*” OR "operative treatment" OR "surgical care" OR "surgical correction" OR "surgical intervention" OR "surgical management" OR "surgical operation" OR "surgical repair" OR "surgical restoration" OR "surgical therap*" OR "surgical treatment*" OR "adverse event*" OR "adverse reaction*" OR C-Reactive Protein OR "creactive protein" OR crp OR "serum c reactive protein" OR "fecal calprotectin")))
Web of Science Core Collection
TS=(("inflammatory bowel disease" OR "crohn disease" OR "crohn's disease" OR "enteritis regionalis" OR "morbus crohn" OR "regional enteritis" OR "regional enterocolitis" OR ileocolitis OR "ulcerative colitis" OR "chronic ulcerative colitis" OR "colitis ulcerosa" OR "colitis ulcerosa chronica" OR "histiocytic ulcerative colitis" OR "mucosal colitis" OR "ulcerative colorectitis" OR "ulcerative procto colitis" OR "ulcerative proctocolitis" OR "ulcerous colitis" OR "colitis gravis")) AND TS=(("electronic nicotine delivery system*" OR E-Cigarette* OR E-Cig* OR vaping OR "e-cigarette smoking" OR "electronic cigarette smoking")) AND TS=((recurrence* OR relapse* OR hospitalization* OR "hospital stay*" OR corticosteroid* OR "adrenal cortex hormone*" OR "adrenal cortical hormone*" OR "adrenal cortical steroid*" OR "adrenal steroid*" OR "adrenal steroid hormone*" OR "adreno cortical steroid*" OR "adreno corticosteroid*" OR "adrenocortical hormone*" OR "adrenocortical steroid*" OR adrenocorticosteroid* OR "cortical steroid*" OR "cortico steroid*" OR corticoid* OR corticosteroid* OR "corticosteroid agent*" OR "corticosteroid calcium" OR "corticosteroid hormone*" OR surgery OR surgeries OR operation* OR "operative intervention*" OR "operative surgery" OR "operative surgical procedure*" OR "operative treatment*" OR "surgical care" OR "surgical correction" OR "surgical intervention" OR "surgical management" OR "surgical operation" OR "surgical repair" OR "surgical restoration" OR "surgical therapy" OR "surgical treatment" OR "adverse event*" OR "adverse reaction*" OR C-Reactive Protein OR "creactive protein" OR crp OR "serum c reactive protein" OR "fecal calprotectin"))
ClinicalTrials.gov
("inflammatory bowel disease" OR IBD OR "crohn disease" OR "crohn's disease" OR "enteritis regionalis" OR "morbus crohn" OR "regional enteritis" OR "regional enterocolitis" OR ileocolitis OR "ulcerative colitis" OR "chronic ulcerative colitis" OR "colitis ulcerosa" OR "colitis ulcerosa chronica" OR "histiocytic ulcerative colitis" OR "mucosal colitis" OR "ulcerative colorectitis" OR "ulcerative procto colitis" OR "ulcerative proctocolitis" OR "ulcerous colitis" OR "colitis gravis") AND ("electronic nicotine delivery system*" OR E-Cigarette* OR E-Cig* OR vaping OR "e-cigarette smoking" OR "electronic cigarette smoking") AND (recurrence* OR relapse* OR hospitalization* OR "hospital stay*" OR corticosteroid* OR "adrenal cortex hormone*" OR "adrenal cortical hormone*" OR "adrenal cortical steroid*" OR "adrenal steroid*" OR "adrenal steroid hormone*" OR "adreno cortical steroid*" OR "adreno corticosteroid*" OR "adrenocortical hormone*" OR "adrenocortical steroid*" OR adrenocorticosteroid* OR "cortical steroid*" OR "cortico steroid*" OR corticoid* OR corticosteroid* OR "corticosteroid agent*" OR "corticosteroid calcium" OR "corticosteroid hormone*" OR surgery OR surgeries OR operation* OR "operative intervention*" OR "operative surgery" OR "operative surgical procedure*" OR "operative treatment*" OR "surgical care" OR "surgical correction" OR "surgical intervention" OR "surgical management" OR "surgical operation" OR "surgical repair" OR "surgical restoration" OR "surgical therapy" OR "surgical treatment" OR "adverse event*" OR "adverse reaction*" OR C-Reactive Protein OR "creactive protein" OR crp OR "serum c reactive protein" OR "fecal calprotectin")
MedNar
("inflammatory bowel disease" OR IBD OR "crohn disease" OR "crohn's disease" OR "enteritis regionalis" OR "morbus crohn" OR "regional enteritis" OR "regional enterocolitis" OR ileocolitis OR "ulcerative colitis" OR "chronic ulcerative colitis" OR "colitis ulcerosa" OR "colitis ulcerosa chronica" OR "histiocytic ulcerative colitis" OR "mucosal colitis" OR "ulcerative colorectitis" OR "ulcerative procto colitis" OR "ulcerative proctocolitis" OR "ulcerous colitis" OR "colitis gravis") AND ("electronic nicotine delivery system" OR E-Cigarette* OR E-Cig OR vaping OR "e-cigarette smoking" OR "electronic cigarette smoking") AND (recurrence OR relapse OR hospitalization OR "hospital stay" OR corticosteroid OR "adrenal cortex hormone" OR "adrenal cortical hormone" OR "adrenal cortical steroid" OR "adrenal steroid" OR "adrenal steroid hormone" OR "adreno cortical steroid" OR "adreno corticosteroid" OR "adrenocortical hormone" OR "adrenocortical steroid" OR adrenocorticosteroid OR "cortical steroid" OR "cortico steroid" OR corticoid OR corticosteroid OR "corticosteroid agent" OR "corticosteroid calcium" OR "corticosteroid hormone" OR surgery OR surgeries OR operation OR "operative intervention" OR "operative surgery" OR "operative treatment" OR "surgical care" OR "surgical correction" OR "surgical intervention" OR "surgical management" OR "surgical operation" OR "surgical repair" OR "surgical restoration" OR "surgical therapy" OR "surgical treatment" OR "adverse event" OR "adverse reaction" OR C-Reactive Protein OR "creactive protein" OR crp OR "serum c reactive protein" OR "fecal calprotectin")
Google Scholar
("inflammatory bowel disease" OR IBD OR "crohn disease" OR "crohn's disease" OR "ulcerative colitis") AND ("electronic nicotine delivery system*" OR E-Cigarette* OR E-Cig* OR vaping OR "e-cigarette smoking" OR "electronic cigarette smoking") AND (recurrence* OR relapse* OR hospitalization* OR corticosteroid* OR "corticosteroid agent*" OR surgery OR surgeries OR operation* OR "operative intervention*" OR "adverse event*" OR "adverse reaction*" OR C-Reactive Protein* OR "fecal calprotectin")

**Table 6:** Risk of bias assessment JBI: Joanna Briggs Institute

Risk of Bias for Case-Control and Cohort Studies (Newcastle-Ottawa Scale)										
Study	Design	Selection	Comparability	Exposure/Outcome	Total Score	Key Reasons for Lost Points				
Sheehan et al., (2023) [9]	Retrospective Matched Case-Control	4/4	2/2	2/3	8	Exposure Ascertainment: Lost 1 point because exposure relied on unstructured electronic health record notes rather than structured, blinded interviews, precluding data on dose, puff frequency, or exact start date.				
Parigi et al., (2025) [13]	Retrospective Postoperative Cohort	4/4	2/2	2/3	8	Adequacy of Follow-up: Lost 1 point because the primary outcome (endoscopic Rutgeerts score) was only available for 81.1% (760/937) of the included cohort, meaning ~19% were missing the primary endpoint.				
Risk of Bias for Cross-Sectional/Observational Studies (JBI Checklist)										
Study	Q1. Inclusion Criteria	Q2. Subjects/Setting	Q3. Valid Exposure Measurement	Q4. Standard Condition Criteria	Q5. Confounders Identified	Q6. Confounders Controlled	Q7. Valid Outcome Measurement	Q8. Appropriate Stats	Total Score	Key Reasons for Lost Points
Malibary et al., (2021) [12]	Y	Y	N	Y	Y	Y	Y	N	6	Lost points on Q3 (Exposure was self-reported via a patient questionnaire subject to recall bias) and Q8 (Statistical analysis for the vaping subgroup was mathematically unstable due to only 3 exposed patients having a 100% flare rate, precluding reliable inference).
Chong et al., (2019) [8]	Y	Y	N	Y	Y	N	Y	N	5	Lost points on Q3 (Self-reported smoking/vaping status), Q6 (Failed to analytically control for confounding; the authors explicitly noted they were unable to separate vaping effects from prior cigarette smoking but did not adjust for it), and Q8 (The exploratory prospective sub-analysis lacked statistical power).
